# Unsupervised Domain Adaptation for Vertebrae Detection and Identification in 3D CT Volumes Using a Domain Sanity Loss

**DOI:** 10.3390/jimaging8080222

**Published:** 2022-08-19

**Authors:** Pascal Sager, Sebastian Salzmann, Felice Burn, Thilo Stadelmann

**Affiliations:** 1 Centre for AI, Technikumstrasse 71, Zurich University of Applied Sciences, 8400 Winterthur, Switzerland; 2 Cantonal Hospital Aarau, AI and Data Science CoE, Tellstrasse 25, 5001 Aarau, Switzerland; 3 ECLT European Centre for Living Technology, 30123 Venice, Italy

**Keywords:** unsupervised domain adaptation, semi-supervised learning, vertebrae detection, vertebrae identification, transfer learning, semantic segmentation, data centrism, deep learning

## Abstract

A variety of medical computer vision applications analyze 2D slices of computed tomography (CT) scans, whereas axial slices from the body trunk region are usually identified based on their relative position to the spine. A limitation of such systems is that either the correct slices must be extracted manually or labels of the vertebrae are required for each CT scan to develop an automated extraction system. In this paper, we propose an unsupervised domain adaptation (UDA) approach for vertebrae detection and identification based on a novel Domain Sanity Loss (DSL) function. With UDA the model’s knowledge learned on a publicly available (source) data set can be transferred to the target domain without using target labels, where the target domain is defined by the specific setup (CT modality, study protocols, applied pre- and processing) at the point of use (e.g., a specific clinic with its specific CT study protocols). With our approach, a model is trained on the source and target data set in parallel. The model optimizes a supervised loss for labeled samples from the source domain and the DSL loss function based on domain-specific “sanity checks” for samples from the unlabeled target domain. Without using labels from the target domain, we are able to identify vertebra centroids with an accuracy of 72.8%. By adding only ten target labels during training the accuracy increases to 89.2%, which is on par with the current state-of-the-art for full supervised learning, while using about 20 times less labels. Thus, our model can be used to extract 2D slices from 3D CT scans on arbitrary data sets fully automatically without requiring an extensive labeling effort, contributing to the clinical adoption of medical imaging by hospitals.

## 1. Introduction

Fine-tuned AI-driven software tools allow an automated analysis of digital images and play a highly relevant role in different industries, especially in healthcare [1]. Computed tomography (CT) images provide accurate information about structural anatomy, morphology, as well as quantitative and qualitative composition of body parts [2]. They usually consist of multiple 2D slices stacked as a batch and form therefore a 3D data set. CT scan processing often relies on the feature extraction capabilities of modern deep learning architectures [3], and many modern deep learning systems process 3D scans as a whole [4,5]. An alternative to 3D scan processing is to extract representative 2D slices first [6], which, for example, can be used for preoperative surgical assessment as well as to examine metabolic, pulmonary, and neurological diseases [7,8]. Such relevant 2D slices of the upper body are usually identified based on their relation to the spine [8,9,10] and can either be extracted manually [10] or automatically, where automatic systems therefore need to be able to recognize the vertebrae and extract the slice containing the relevant information [11]. Usually, this requires knowledge of vertebrae locations, i.e., manually created labels for a multitude of 3D CT volumes, to train respective systems.

**Figure 1:**
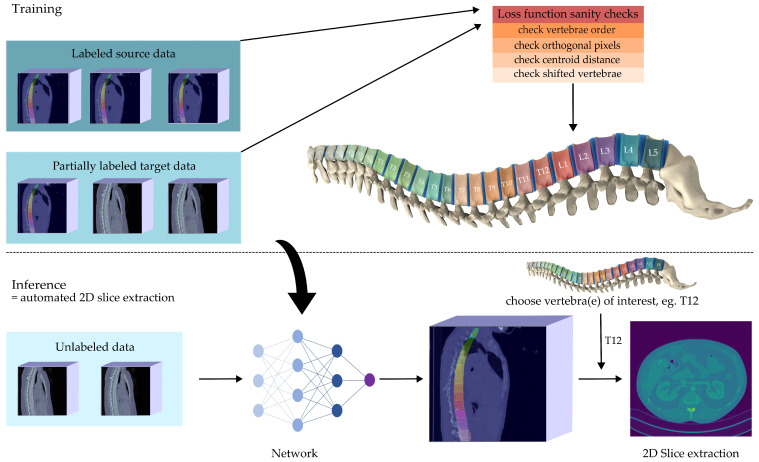
Visual abstract of our work: We train a vertebrae Detection and Identification module simultaneously on a publicly available data set (source domain) and a second custom data set (target domain). We require only a few labels from the custom data set. With the help of a loss function that is inspired by anatomical domain knowledge the proposed model is able to identify vertebrae centroids with state-of-the-art performance, reducing the need for target-domain labels by a factor of 20. We see its main application within ML-pipelines to extract representative 2D slices out of 3D volumes, representing a step towards fully automated systems for downstream 2D slice analysis.

In this paper, we present an approach to identify vertebrae of the spine automatically without the need of excessive labeling of own data (or even no labels at all), thereby heralding a data-centric approach [12] based on un- or semi-supervised learning [13]. To this end, our contribution is the development and evaluation of a novel method that requires no labels at all to achieve reliable vertebrae detection and identification and, if given less than 5% of the labels we perform on par with comparable supervised approaches. Thus, our approach reduces the labor-intensive labeling effort that can hinder applicability in medical institutions. An overview of our approach is given in Figure 1. The quality of our results allows the extraction of representative 2D slices from 3D volumes within an automated machine learning (ML) pipeline.

The remainder of the paper is organized as follows: In Section 2, we review the related work and argue why we build upon the work of McCouat and Glocker [14]. In Section 3, we explain how we extended the “Detection” module with post-processing and propose a new unsupervised loss function for the “Identification” module. In Section 4, we present the results of our method in detail and show how well vertebrae can be detected and identified with only a few labels. In Section 5, we conclude that our method facilitates the application in medical institutions, as very good results are obtained with an order of magnitude fewer labels than comparable methods require. Furthermore, we identify limitations and suggest future research directions.

## 2. Related Work

The detection and identification of vertebrae is well studied. However, many methods for vertebrae identification make prior assumptions. For example, Zhou et al. [15] assume that the first sacrum vertebra (S1) is within the image while Yi et al. [16] assume that always the same vertebrae are visible. The model of Altini et al. [17] on the other hand requires manual input with meta-information about the first visible vertebra. Other approaches make assumptions about the shape of the spine [18] and therefore do not work well in pathological cases where the spine is deformed. In contrast, this work does not impose such assumptions, enabling processing of a broad range of CT scans even if the images only contain cropped parts of the spine.

Predicting the vertebra centroids directly (i.e., as a regression task) often leads to poor results [19]. Therefore most approaches turn the regression problem into a dense classification problem [14,16,19]. Earlier approaches used classical machine learning models such as random forests to identify vertebra centroids [19] while more recent approaches achieve better results using convolutional neural networks (CNNs). For example, Yang et al. [20] use an encoder-decoder architecture together with multi-level feature concatenation to locate vertebrae. The extracted centroid probability maps are iteratively improved based on the mutual relation of vertebra centroids. Liao et al. [21] achieve state-of-the-art results using a CNN to detect the positions of the centroids, combining it with a recurrent neural network (RNN) to capture the ordering of the vertebrae.

McCouat and Glocker [14] obtained similar results using two separate U-Nets [22] for detecting and identifying vertebrae. Their data set consists of 3D CT scans with labels for the vertebrae centroids. Initially, these sparse labels are converted to dense labels. Then the “Detection” module, the first in the two-stage approach, detects the spine within the 3D volume. To enable training with limited computational resources the 3D volumes are divided into smaller patches. Each of these patches is fed into a 3D U-Net that segments the vertebrae from the background. Once the spine is located the relevant region is extracted from the 3D volume and processed by the second module.

This second stage is the “Identification” module that maps pixels to the corresponding vertebrae. For this purpose, a 2D U-Net is used. The model does not classify each pixel but produces a continuous value for each pixel. Rounding this continuous value results in an integer which is associated with a vertebra (e.g., 1 = C1, 2 = C2, ...). Due to the prediction of continuous values per pixel the L1 loss function can be used to capture the order of the vertebrae. The Identification module predicts a value for each pixel, even if that pixel depicts background and not a vertebra. Since the Detection module classifies the background pixels as 0 the output of the Identification module is multiplied by the output of the Detection module yielding the prediction without background. Finally, the predicted dense labels are converted back to sparse labels by calculating their median position.

In this work, we extend this approach from McCouat and Glocker [14] with unsupervised domain adaptation (UDA) methods. We extend the Detection module with post-processing and the Identification module with a new Domain Sanity Loss (DSL) based on “sanity checks”. We build upon their work for the following reasons: (i) The average distance between the predicted and the actual vertebrae centroids is small and considered state-of-the-art; (ii) the models are pure CNN architectures which can be easily extended within the framework of deep learning [23]; (iii) no assumptions are made about neither the shape of the spine nor the visible vertebrae. This way, the model is adapted to the target data, which is considerably easier to train in our experience than the alternative of adapting the data to the model [24].

## 3. A Method for Unsupervised Domain Adaptation of CT Scans of the Spine

The method of McCouat and Glocker [14] performs well on labeled data sets. However, performance is poor when the trained model is applied to other data sets on which it has not been trained (c.f. Section 4). To process data from other domains, we extend the two modules. The Detection module is extended with post-processing, while the Identification model is trained with a new DSL loss function. The proposed UDA training procedure for the Identification module leverages publicly available labels and helps the model to adapt to a second data set even without labels. Since we adapt the knowledge learned on one domain to another, we refer to the first domain as the source and the second as the target domain. Our extensions only affect the training process, while the network architecture remains unchanged.

### 3.1. Detection Module

In accordance with [14] we divide the 3D volumes of the source and target data set into smaller patches of size [80×80×96] and process them with a 3D U-Net. An advantage of processing patches instead of the entire 3D volume is that the model can be trained with limited computational resources. The sparse annotations (i.e., centroid positions of vertebrae) are converted into dense annotations (i.e., pixel-level labels) [14]. Pixels depicting a vertebra are labeled as 1, and pixels depicting background as 0. Adam [25] is used with a learning rate of $1\times 10^{-3}$ during training to minimize a binary cross entropy (BCE) loss. The model is trained with a batch size of 16 samples for 70 epochs. After training the model labels pixels either as spine or background. Thus, this module can locate the spine in a 3D volume.

In contrast to [14], we post-process the predictions of our model. This post-processing is helpful because it can be hard for the model to detect parts of the spine in small patches. Processing patches is considered more difficult than processing the entire CT scan because of the lack of context provided by the surrounding pixels. After all patches of a scan are predicted we conduct a connected component analysis on the 3D volume. It identifies all connected groups of pixels that are labeled as spine. Since the spine consists of many pixels, it is retained as the biggest component while smaller components are discarded as artefacts. To remove only artefacts and not the spine from the prediction we weigh the BCE loss by a factor of 1.0 for the spine and 0.1 for the background. By doing so, the spine is detected as a single component with very high accuracy and not removed as an artefact.

### 3.2. Identification Module and Domain Sanity Loss

The Identification module processes patches of the size [8×80×320] in a 2D U-Net as in [14]. These patches have a large field of view of 80×320 pixels along the sagittal plane thus allowing identification of vertebrae. As conducted in the Detection module, the sparse annotations are converted to dense annotations, background is labeled as 0 and the vertebrae with integers in ascending order (i.e., 1 = C1, 2 = C2, ⋯, 26 = S2).

**Figure 2:**
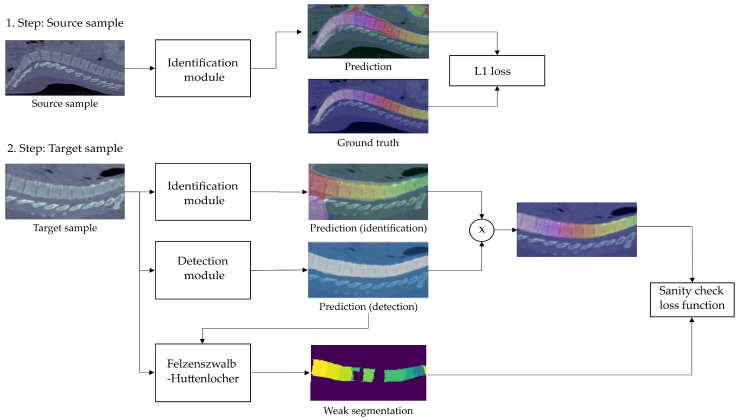
2-way training process of the Detection module: In step one, L1 distance is used to calculate the loss of a mini-batch of source domain samples. In step two, several “sanity checks” (see Figure 3 for an overview) are calculated to form the loss of a mini-batch of target-domain data. The sanity-check-based DSL loss only considers spine pixels by multiplying the output of the Identification module with the output of the Detection module and employs the Felzenszwalb-Huttenlocher algorithm [26] to create a weak segmentation mask of vertebrae location in an unsupervised way (c.f. Section 3.2).

In contrast to [14], we extend this module with an UDA method. Our proposition is based on a novel training process that instead of processing only samples from the source domain is alternatingly feeding mini-batches from the source and target domain into the model. The intuition behind this is that samples from the source domain teach the model vertebrae identification while samples from the target domain help to adapt to the target data set. This 2-way training procedure is shown in Figure 2.

**Figure 3:**
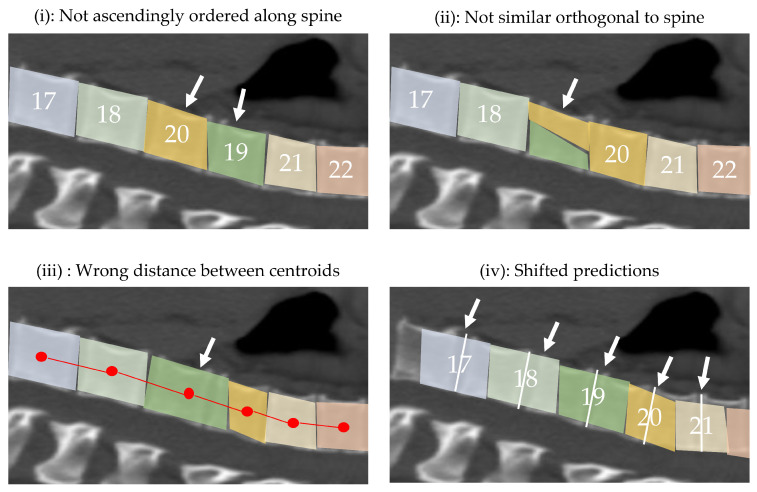
Visual representation of the sanity checks performed by the proposed Domain Sanity Loss (DSL) function; the displayed cases show failures for each check, indicated by the white arrows. Specifically, the DSL loss checks for (**i**) monotonous ascend of predicted vertebrae numbers along the spine; (**ii**) all spine pixels in one column of the image having the same vertebra number; (**iii**) predicted vertebrae centroids having a reasonable distance to each other, based on average distances from the literature; and (**iv**) predictions not being shifted along the spine, based on an unsupervised weak segmentation of the vertebrae (c.f. Figure 2).

In the first phase, since the source data samples have labels, a supervised L1 loss function is used as suggested by [14]. By predicting continuous values and not label probabilities, this function is able to measure the distance to the ground truth vector rather than merely checking for equality (e.g., prediction C2 is better than prediction C3 for label C1) and thus considers the order of the vertebrae. However, since no labels are available for the target data set no supervised loss function can be used in the second phase. Therefore, we propose the Domain Sanity Loss (DSL) based on “sanity checks” as introduced and illustrated in Figure 3.

The DSL loss is with its four checks purely based on anatomically induced invariances that hold true even for severely deformed spines and hence need no corresponding human-provided labels for any image. As these invariances only apply to pixels belonging to the spine, we multiply the model output with the prediction of the previous Detection module and thereby set all pixel values that do not belong to the spine to zero. In the following, we denote this prediction with removed background as $\hat{y}$: a matrix of the same shape as an input image with the predicted vertebra number for spinal pixels (i.e., 1 = C1, 2 = C2, ...) and 0 otherwise. We denote *i* as row and *j* as column indices of $\hat{y}$ and $n_{row}$ and $n_{col}$ as the number of pixels per row and column of the sagittal plane respectively. Furthermore, we define the identification function for boolean values as

(1) $$1_{b}\left(x\right)=\left\{\begin{array}{cc} 1 & \mathrm{if}\;x\;\mathrm{is}\;\mathrm{true}\\ 0 & \mathrm{otherwise} \end{array}\right.$$

The first term $s_{1}$ of the DSL loss function (c.f. Equation (6) at the end of this subsection) evaluates whether the *vertebrae are sorted in ascending order along the spine*. For a correct prediction, the per-pixel values in $\hat{y}$ along the longitudinal axis must be sorted in ascending order (c.f. Figure 3(i)). We implement this by comparing each predicted pixel ${\hat{y}}_{i,j}$ with a version of the same prediction ${\hat{y}}_{i,j+s}$ shifted to the right by *s* pixels. Thereby we evaluate if a pixel shifted to the right of any given pixel still gets the same or a higher prediction. In doing so, we check whether pixels are sorted ascending from the left to the right. All pixels that do not fulfill this criterion lead to an increase in the loss value. We ignore the pixel values that get shifted outside of the range of the original prediction which is why we only sum up the pixels column wise until $n_{col-s}$. We define the first loss term as

(2) $$s_{1}\left(\hat{y}\right)=\frac{1}{n_{pix}}\sum_{s=1}^{n_{shift}}\sum_{i=1}^{n_{row}}\sum_{j=1}^{n_{col}-s}1_{b}\left({\hat{y}}_{i,j}-{\hat{y}}_{i,j+s}\geq 0\right)$$

where $n_{shift}$ is the maximum range of shift, and $n_{pix}$ the number of pixels in ${\hat{y}}_{i,j}$. Empirically, we found that shifting values s>30 do not enhance the result anymore. We therefore define $n_{shift}=30$ and thus compare the order of the vertebrae only locally which leads to higher computational efficiency. We divide the number of pixels that violate this constraint by the number of total pixels $n_{pix}$ and therefore $s_{1}\left(\hat{y}\right)$ captures the percentage of spinal pixel for which the anatomical order of the vertebrae is not correct.

The second term $s_{2}$ of the loss function checks whether the *pixel values orthogonal to the spine are identical*. For this we analyze the pixels that are differently labeled along the sagittal axis (c.f. Figure 3(ii)). We assume the median value of each column *j* of ${\hat{y}}_{i,j}$ as label of that column and compare it to all values in that column. We denote $v_{j}$ as the column vector of ${\hat{y}}_{i,j}$ at index *j*. Furthermore, we define a function $median(v_{j})$ which calculates the median of a column vector $v_{j}$. We assume that the spine is more or less parallel to it (rotation can be checked easily by pre-processing, if necessary). We define the second loss term as

(3) $$s_{2}\left(\hat{y}\right)=\frac{1}{n_{pix}}\sum_{i=1}^{n_{row}}\sum_{j=1}^{n_{col}}1_{b}\left(\right|{\hat{y}}_{i,j}-median\left(v_{j}\right)\left|>0\right)$$

For each column, we sum up the number of pixels that are labeled differently than the median and divide this sum by the total number of pixels. Thereby we obtain a factor that indicates how consistent the vertebrae per column and thus orthogonal to the spine are.

The third term $s_{3}$ of the DSL loss function evaluates the *distance between the centroids of the predicted vertebrae* (c.f. Figure 3(iii)). We define the distance between vertebra *i* and *j* as $\delta_{i,j}$. We denote the average distances of vertebrae as taken from Busscher et al. [27] as ${\bar{\delta }}_{i,j}$. We denote the upper bound of the summation as $n_{vert}=25$, which is the number of vertebrae of a spine (26) minus one. The third loss term

(4) $$s_{3}\left(\hat{y}\right)=\frac{1}{n_{vert}}\sum_{i=1}^{n_{vert}}\left|\delta_{i,i+1}-{\bar{\delta }}_{i,i+1}\right|$$

calculates the Euclidean distances between subsequent vertebra centroids and compares it to the gold standard from literature using the L1 loss. If the distance between two vertebrae is equal to the gold standard the loss is 0, otherwise it is bigger than 0. We sum up the distance differences between subsequent vertebrae to the third term $s_{3}\left(\hat{y}\right)$. We therefore perform an explicit sanity check on vertebrae distance and an implicit check on vertebrae size.

The fourth term $s_{4}$ of the loss function checks *whether the predicted vertebrae are not shifted*. So far it has only been verified whether the spine is anatomically correctly detected. However, the spine itself may be slightly displaced within the image (c.f. Figure 3(iv)). To detect shifts we make use of a weak segmentation mask which is constructed as follows: First, the input scan (and not the mask) is multiplied by the prediction of the Detection module to extract the spine, followed by setting all pixels below an intensity threshold of 180HU to 0 in order to emphasize the edges. We then use the Felzenszwalb-Huttenlocher algorithm [26] to predict a segmentation mask of the vertebrae in a unsupervised manner. As this mask is relatively imprecise it is referred to as a weak mask wm. The predicted mask is further improved by heuristically filtering out components that cannot correspond to a vertebra (e.g., wrong shape) and by merging components that are enclosed in one another.

The weak mask wm has the same shape as the prediction $\hat{y}$. Each pixel in the weak mask is assigned to a connected component $c_{k}\in wm$. Each $c_{k}$ has a set of row $c_{k_{i}}$ and column $c_{k_{j}}$ coordinates which pairwise represent all pixels of a component. The intuition behind this fourth loss term is that the prediction $\hat{y}$ should have the same label at the coordinates of pixels that belong to the same connected component $c_{k}$. For each connected component $c_{k}$ we extract from ${\hat{y}}_{i,j}$ the values at the positions $\left(i,j\right)\in \left(c_{k_{i}},c_{k_{j}}\right)$ and define this operation as $v(\hat{y},c_{k})$. Furthermore, we define u(x) which returns the number of unique values in a set *x*. Based on our definition $u(v\left(\hat{y},c_{k}\right))$ returns the number of unique values within ${\hat{y}}_{i,j}$ at the coordinates $\left(c_{k_{i}},c_{k_{j}}\right)$ of a connected component $c_{k}$.

Per connected component $c_{k}$ the pixels in the prediction $\hat{y}$ should be labeled identically and thus $u(v\left(\hat{y},c_{k}\right))$ should return 1. If multiple labels are predicted at the positions of a connected component, $u(v\left(\hat{y},c_{k}\right))$ returns a value greater than 1. The fourth part of our DSL loss function sums up the number of inconsistent labels per connected component:

(5) $$s_{4}\left(m,\hat{y}\right)=\frac{1}{n_{c}}\sum_{c_{k}\in wm}u\left(v\left(\hat{y},c_{k}\right)\right)-1$$

The domain-specific DSL loss function therefore consists of four sanity checks that penalize anatomical inconsistencies. To obtain the DSL loss value, we sum the four loss terms:

(6) $$L\left(m,\hat{y}\right)=c_{1}\cdot s1\left(\hat{y}\right)+c_{2}\cdot s2\left(\hat{y}\right)+c_{3}\cdot s3\left(\hat{y}\right)+c_{4}\cdot s4\left(m,\hat{y}\right)$$

where the constants $c_{s}$ are scaling values that we found experimentally to work well when set to $c_{1}=20$, $c_{2}=1$, $c_{3}=1/40$, and $c_{4}=1/100$ as they bring the four loss parts to an approximately similar scale. To optimize this loss, we use Adam [25] as optimizer with a learning rate of $5\times 10^{-4}$. The model is trained for 100 epochs with a batch size of 32 samples.

### 3.3. Data Sets

We use the BioMedIA Spine data set [28] as source data set. It consists of 242 spine-focused CT-scans of 125 patients with varying types of pathologies. In most scans, the view is limited to 5–15 vertebrae, while only a few scans depict the entire spine [19]. The scans differ significantly in terms of image noise, physical resolution, and vertical cropping [18]. Each scan is labeled with point-annotations of vertebrae centroids that are extended to dense labels using the approach outlined in [14]. The data set provides a predefined split which is used for training and testing.

To test the proposed unsupervised domain adaptation schema for vertebrae detection and identification, the COVID19-CT data set [29,30] with 1000+ scans from patients with confirmed COVID-19 diagnosis is used. The scans are composed of 16-bit grayscale images with a size of 512×512 pixels [29]. Most of the scans have an inter-axial distance between 0.5 and 1.5 mm. A radiology experienced physician labeled the vertebra centroids of a random subset with 30 scans, of which 20 are used as a test set and 10 labeled scans optionally together with the remaining scans as training set.

Similar to [14], we divide all samples into smaller patches. To train the Detection module on the source data set we extract 10 patches of the size [80×80×96] from random positions out of each scan. Thereby we ensure that at least 8 out of the 10 patches contain parts of the spine. Since the Detection module is not trained on the target data set, only patches from the labeled source data set are needed. For testing on the source as well as the target data set, we divide the entire scan independent of the position of the spine into patches of the size [80×80×96].

For the training of the Identification module, we extract 300 patches with a shape of [8×80×320] per sample. If labels exist we ensure that each patch contains at least one vertebra. If no labels exist we use the output of the Detection module to locate the spine and extract patches out of this region. For testing, the entire scan is again divided into patches.

## 4. Results

In the following three subsections, we analyze our Detection and Identification module experimentally, comparing them to prior and related work.

### 4.1. Detection Results with and without Post-Processing

**Figure 4:**
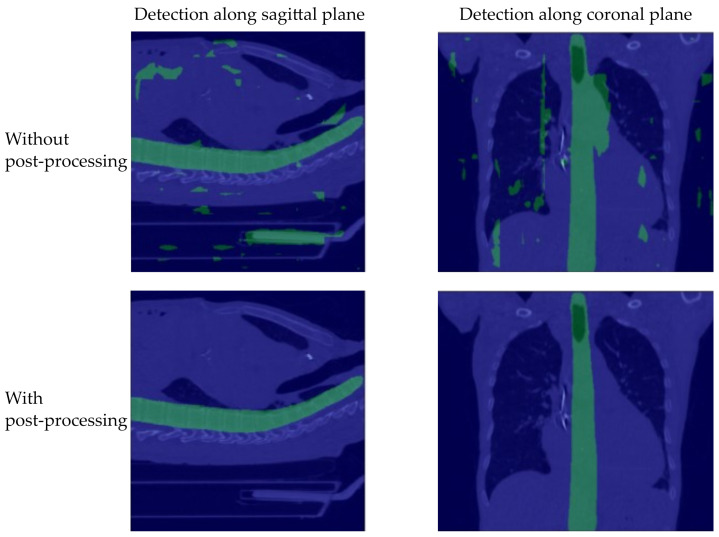
Four randomly selected samples from the target data set (COVID-19 CT) with overlayed predictions for the spine detection with (**bottom row**) and without (**top row**) post-processing. To provide a better grasp of the post-processing’s effect, we visualize all predictions within the 3D mask along the sagittal plane (**left**) and along the coronal plane (**right**).

The Detection module detects the spine within the 3D volume well. However, without post-processing many false-positive predictions (i.e., prediction “spine” instead of “background”) lead to bad results, especially on the target data set without labels (c.f. Figure 4). A possible reason for this is that the model is trained only on small patches of the original volume. Therefore, the model only learns to identify parts of vertebrae and not how a whole spine looks like. An indication for this is that false negatives are often detected in places with cubic shapes, for example, the bed on which the patient is lying. However, since these false predictions consist of far fewer connected pixels than the entire spine our post-processing is able to successfully remove these artefacts.

To highlight how our post-processing improves performance we calculate various metrics. However, these metrics must be interpreted with caution for two reasons: (i) Generated dense annotations, which are calculated based on average sizes of vertebrae bodies, are used as ground truth; such annotations are by design less accurate than, for example, carefully hand-crafted segmentation masks. (ii) The performance is calculated on the whole volume and not on cropped samples as is conducted in [14]. Since the cropped samples have a much higher proportion of pixels representing the spine these results are not directly comparable. However, the published results of [14] correspond roughly with the performance of our model without post-processing as both are based on the same method.

Table 1 shows the results of the Detection module. The accuracy, recall, intersection over union (IoU), and dice-score are calculated for the source data set (BioMedIA) and for the target data set (COVID19-CT). The proposed post-processing clearly improves the performance. For example, the IoU of pixels representing vertebrae in the source data set improves from 67.4% to 78.7%, which is a 16.8% relative improvement. The improvement on the target data set is even more noticeable. Using post-processing on the target data set, IoU improves from 46.4% to 79.1%. While the model without post-processing is considered not accurate enough to detect the vertebrae on the target data set, the model with post-processing is suitable for the subsequent vertebrae identification.

### 4.2. Identification Results per Spinal Pixel

We trained the Identification module in three different setups: (i) A first model is trained without UDA and only using source labels, corresponding to the same method as proposed in [14]; (ii) a second model is optimized with the proposed DSL loss of Section 3.2; (iii) a third model is given ten random training samples plus their labels from the target data set, used in the same fashion as source samples.

**Figure 5:**
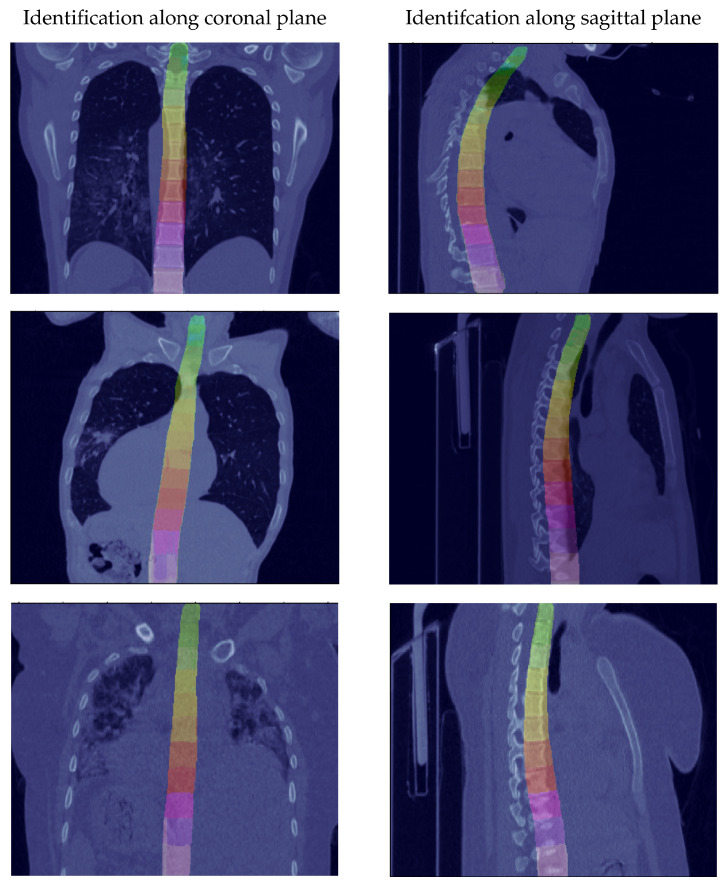
Random samples of prediction from the Identification module on the target data set (COVID-19 CT), showing satisfactory results even when the spine is not well aligned on the coronal and sagittal axis.

To compare the models with and without UDA, the classification accuracy per pixel is measured. The accuracy is determined by dividing the number of correctly classified pixels by the total number of pixels. Thereby, only the pixels belonging to the spine are taken into account and the background is ignored. As shown in Table 2, the model without UDA (i) achieves a classification rate of 13.3% on the target data set. The model with UDA (ii) achieves an accuracy of 61.4%. This corresponds to a relative improvement of 462.7% and demonstrates the effectiveness of the proposed approach. If additionally ten samples from the target data set are labeled (iii), the identification rate further improves to 74.2%. We display some predictions in Figure 5. This visualization demonstrates that the vertebrae are well recognized.

### 4.3. Identification Results per Vertebra

The results described so far refer to the classification accuracy per pixel. However, the goal is to identify the vertebra centroids and therefore the obtained dense predictions must be converted back into sparse centroid predictions. This is conducted by calculating the median of the dense predictions as described by [14], thereby ignoring outliers in the pixel-level prediction by virtue of the median. The results of the centroid predictions are shown in Table 3. We define the identification rate “ID” as the number of correctly identified vertebrae divided by the total number of vertebrae. We count an identification as correct if the predicted centroid is no more than 20 mm away from the ground truth. We use 20 mm as this is an often used reference distance [14,18,21] and therefore makes our method comparable to other approaches. Only the results on thoracic vertebrae are compared since vertebrae from other regions are underrepresented in the COVID-19 CT data set (CT scans can be classified into regions depending on the body part they are taken from. Well-known areas are the cervical region (neck level), the thoracic region (chest level) and the lumbar region (pelvis level). For state-of-the-art AI models the thoracic region is the most challenging one because only a middle section of the spine is visible in these scans and therefore vertebrae cannot be counted from the first cervical vertebra (C1), respectively the last sacrum vertebra (S2)).

As before, “our method” corresponds to the model proposed in [14] with additional UDA extensions. The results obtained with this model on the BioMedIA source data set are less accurate than those of the original model without UDA. A reason is that our model was optimized for the target data set only. Furthermore, by using domain adaptation a performance loss on the source data set was consciously accepted in exchange for better results on the target data set. If ten labels from the target data set are added during training the model is superior to the original one on the source data set. Reasons for this are that (i) the post-processing of the Detection module leads to better identification of the spine and (ii) that the COVID-19 CT data set contains a lot of samples from thoracic vertebrae and thus the model is more optimized for this region.

When analyzing the results on the COVID19-CT data set the effectiveness of the proposed domain adaptation is evident. When the model is trained without UDA, only 45.6% of the vertebrae are correctly classified on the target data set. With the proposed domain adaptation methods, the classification rate increases to 72.8%. A comparison with state-of-the-art results on the BioMedIA data set shows (though being unfair because of the different data sets used to achieve the respective numbers) that this is only 11.2 pp. less accurate than the results of Liao et al. [21] and only 7 pp. less accurate than the results of McCouat and Glocker [14], which both trained their model with labels. If ten labeled target samples are added to the training set, an identification rate of 89.2% is achieved. This is 5.2 pp. better than the best results reported so far for the BioMedIA data set. Of course, the comparability of these remarks is limited because the data sets are different, but it underlines that the performance of our method with semi-supervised domain adaptation is remarkable.

## 5. Conclusions

In this paper, we presented a method to find vertebrae centroids on unlabeled CT data sets, proposing a novel un- and semi-supervised domain adaptation method based on the Domain Sanity Loss function that achieves state-of-the-art results with orders of magnitudes less labels than previous methods. The detection and identification of vertebrae is important, for example, to extract 2D slices at predefined levels from 3D CT scans. Compared to existing state-of-the-art systems our method has the advantage of requiring much fewer labels while obtaining comparable results. For example, in clinical practice, the BioMedIA [28] data set could be used as source data set and be combined with a custom target data set. Our proposed UDA approach would only require the creation of approximately ten labels of the custom data set, whereas a supervised approach might require several hundred labels. Since less labor-intensive labeling is necessary the transfer of the method to other medical applications and facilities is easier and more cost-efficient.

The main drawback of our method is that it requires more computational resources. While supervised methods use one data set, our UDA method requires a source and a target data set. Using an NVIDIA V100 GPU, training takes about 2 days. However, comparable results with an ID rate of over 86% can be achieved after 16.5 h (with 35 instead of 100 epochs). Thus, the training takes slightly more than twice as long as the original method from McCouat and Glocker [14]. Inference, on the other hand, is identical except for the additional post-processing and therefore takes about the same amount of time.

### 5.1. Discussion

Specifically, pixel-level classification is often employed in the medical field [4,5,6]. Training such models in a supervised manner requires labels. Depending on the specific task, labeling a single 3D scan on the pixel level can take an expert up to two weeks [32]. Considering that many applications require several hundred samples, one can conclude that labeling a complete data set is almost prohibitively labor-intensive [33,34], setting harsh limits to AI democratization. Alternatively, representative 2D slices can be used for various applications (c.f. Section 1). These 2D slices are less time-consuming to label, since they are only a cut-out of the 3D data. Thus, not only does our method for extracting 2D slices require very few labels, but it can reduce the labeling effort of downstream ML pipelines because representative 2D slices instead of 3D data can be processed in subsequent systems.

On the COVID-19 CT data set, 89.2% of all vertebra centroid predictions are identified correctly which is in line with (in fact, beyond) the state-of-the-art on other data sets. The mean deviation of the predicted centroid to the ground truth centroid is 8.1 mm. However, this distance is measured in the 3D space. Considering the task of extracting 2D slices the deviation is even smaller because only the error in one direction of the 3D space is relevant. In rare cases vertebrae can be mistaken and the deviation is much bigger, leading to a standard deviation of 20.3 mm. Depending on the application, such wrong predictions can simply be filtered out by analyzing the content of the 2D slice as conducted by [11]. However, since this is application-dependent such post-processing is out of scope of our work.

### 5.2. Limitations and Future Work

The proposed UDA method with DSL loss works very well on our target data set. A limitation, however, is that the fourth loss component $s_{4}$ relies on reference distances between subsequent vertebrae from the literature. Therefore, it is assumed that our approach works worse for patients which do not comply with these reference values (e.g., children). A second limitation is uncommon spinal constellations. In very rare cases, for example, patients may have an additional lumbar vertebra L6, a lumbalizated S1, or a sacralizated L5 as normal deviations to the standard spine. Since these constellations are not included in our label set, they therefore cannot be detected.

In principle, our proposed UDA method and a DSL loss based on domain-specific sanity checks is applicable to other domains and problems as well, even outside of medical image processing. For example, we started experimenting with DSL losses for symbol recognition in document analysis tasks [35]: We calculated statistics of symbols such as their size and orientation, and built DSL losses to ensure that the predictions per page comply with these statistics. From the preliminary experiments, we learned that DSL losses will not work well if the data contains a lot of variation which cannot be specified in the loss function. Furthermore, we found that in this use-case a pre-training is necessary, otherwise the predictions deviate too much from the statistics which hinders the learning process.

With respect to this work, we see further research potential (i) on optimizing performance for patients with a smaller spine and (ii) on reliably detecting and correcting incorrect predictions. The issues for patients with a small spine could be remedied either by using other reference values or by adapting the loss component $s_{4}$ to work with ratios instead of absolute distances. Incorrect predictions, on the other hand, could be detected with statistical methods regarding the centroids or by analyzing the corresponding 2D slice on the transversal plane.

On a more general perspective, the DSL loss is considered complementary to process unlabeled data and could serve as a general domain adaptation method. For example, specifying a framework that derives statistics about sizes and relations of objects from the data set and uses them as sanity checks in the loss function could be helpful for various applications.

## Figures and Tables

**Table 1:** Performance evaluation of the Detection module with the highest score for each metric and data set in bold. For each metric, the overall performance for the whole 3D scan and for the vertebrae with ignored background is reported. The positive effect of our proposed the post-processing is visible on the source and the target data sets.

BioMedIA (Source Data Set)		
Metric	without Post-Processing	with Post-Processing
Accuracy (overall)	99.2%	**99.5**%
Recall (overall/vertebrae)	99.2%/**94.3%**	**99.5%**/94.1%
IoU (overall/vertebrae)	98.3%/67.4%	**99.0%**/**78.7%**
Dice (overall/vertebrae)	99.2%/80.2%	**99.5%**/**88.0%**
**COVID-19 CT (Target Data Set)**		
**Metric**	**without Post-Processing**	**with Post-Processing**
Accuracy (overall)	99.6%	**99.9**%
Recall (overall/vertebrae)	99.6%/**95.1%**	**99.9%**/**95.1%**
IoU (overall/vertebrae)	99.2%/46.4%	**99.8%**/**79.1%**
Dice (overall/vertebrae)	99.6%/63.0%	**99.9%**/**88.0%**

**Table 2:** Classification rate on the COVID19-CT data set for the three trained models with the best classification rate in bold. The effectiveness of un- and semi-supervised domain adaptation is striking.

Classification Rate on COVID-19 CT (Target Data Set)		
Our Method without UDA	Our Method	Our Method (with 10 Labels)
13.3%	61.4%	**74.2%**

**Table 3:** Detection result per vertebra with the best score for each metric and data set in bold. The upper part of the table displays the results on thoracic scans of the source data set, the lower part the results on the target data set. The column “ID” gives the identification rate, column “Mean” reports the average distance to the ground truth centroid in mm and column “Std” gives the standard deviation in mm.

Thoracic Vertebrae BioMedIA (Source Data Set)			
Method	ID	Mean	Std
Chen et al. [31]	76.4%	11.4 mm	16.5 mm
Liao et al. [21]	**84.0%**	7.8 mm	10.2 mm
McCouat and Glocker [14]	79.8%	6.6 mm	7.4 mm
Our method	67.0%	8.4 mm	8.7 mm
Our method (with 10 labels)	80.1%	**6.2 mm**	**7.2 mm**
**Thoracic Vertebrae COVID-19 CT (Target Data Set)**			
**Method**	**ID**	**Mean**	**Std**
Our method without UDA	45.6%	17.4 mm	24.2 mm
Our method	72.8%	11.1 mm	20.8 mm
Our method (with 10 labels)	**89.2%**	**8.1 mm**	**20.3 mm**

## Data Availability

We publish our code on https://github.com/sagerpascal/uda-vertebrae-identification (accessed on 17 August 2022) together with a detailed description of how the data sets as well as the annotations can be accessed.
